# Schwannoma of the Ankle: A Case Report of a Rare Clinical Entity

**DOI:** 10.7759/cureus.72753

**Published:** 2024-10-31

**Authors:** Pedro Ribeiro, Ana Esteves, Joana Monteiro Pereira, Júlio Marinheiro

**Affiliations:** 1 Orthopedics, Unidade Local de Saúde do Tâmega e Sousa, Porto, PRT

**Keywords:** foot, mri, nerve sheath tumors, schwannoma, soft tissue tumors, superficial peroneal nerve

## Abstract

A schwannoma is a soft tissue benign tumor that originates from Schwann cells of the peripheral nerve sheath. It is uncommon for it to occur in the foot. The tumor usually has an indolent presentation with a delayed diagnosis that may lead to irreversible nerve damage. The symptoms are related to the compression of the nerve due to the mass effect of the lesion. The authors report a case of a 62-year-old female with a history of breast cancer who was referred to the orthopedics department with pain in the dorsum of the right foot and a positive Tinel sign in the trajectory of the superficial peroneal nerve. The diagnosis of schwannoma of the superficial peroneal nerve was made, and the patient underwent surgery with complete resolution of symptoms. No deficits or recurrences were observed during the two-month follow-up period.

The purpose of this report is to draw attention to the high index of suspicion and the need for a correct diagnosis for optimal clinical results.

## Introduction

Schwannoma, or neurilemmoma, is a benign tumor encapsulated by epineurium originating from Schwann cells of peripheral nerve sheaths, representing 5% of all benign soft tissue neoplasms and being the most common benign nerve sheath neoplasm [1,2]. Usually, it is an isolated and well-encapsulated tumor and is described in a number of locations. These tumors can occur in the context of patients suffering from type 2 neurofibromatosis (NF2) and may also be the possibility of trauma being the cause of the schwannoma [2,3]. It has an indolent evolution with painless swelling, usually leading to a late diagnosis with the possibility of irreversible damage to the nerve. The onset of symptoms is usually associated with neural compression and spontaneous pain or after exertion, and paresthesia is the main complaint. A Tinel sign is usually present around the mass [4-6].

The presence of schwannomas in the foot is a rare clinical entity with few cases described in the literature [3,7,8]. The diagnosis requires a clinical examination and an MRI. Simple surgical excision after careful dissection is usually sufficient, as recurrence and malignant transformation rates are low [9,10]. This report aims to describe this rare location of schwannoma and discuss its clinical features, diagnosis, and adequate treatment, as well as to draw the attention of the orthopedic community to this diagnosis.

## Case presentation

**Figure 1:**
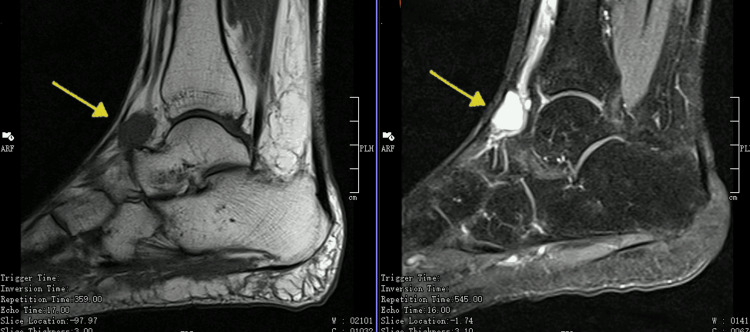
MRI images showing the schwannoma (arrow). The right image is T2-weighted where the schwannoma appears hyperintense, and the image on the left is T1-weighted with the schwannoma appearing isointense MRI: magnetic resonance imaging

The authors present a case of a 62-year-old woman with a medical history of breast cancer in remission. The patient was referred to our outpatient clinic due to pain in the dorsal region of the right foot in the last six months with a palpable mass. When evaluated, she mentioned pain in the dorsum of the right foot, mainly in the second and third rays associated with paresthesia in the same territory. Clinically, she has a palpable mass, firm and without tenderness in the anterolateral region of the ankle. A Tinel sign was present near the mass. For diagnosis, an MRI was requested and showed an oval-shaped, well-defined lesion in the dorsal compartment of the foot involving the lateral cutaneous branch of the superficial peroneal nerve. The schwannoma appears isointense in T1-weighted images and hyperintense in T2-weighted images (Figure 1).

**Figure 2:**
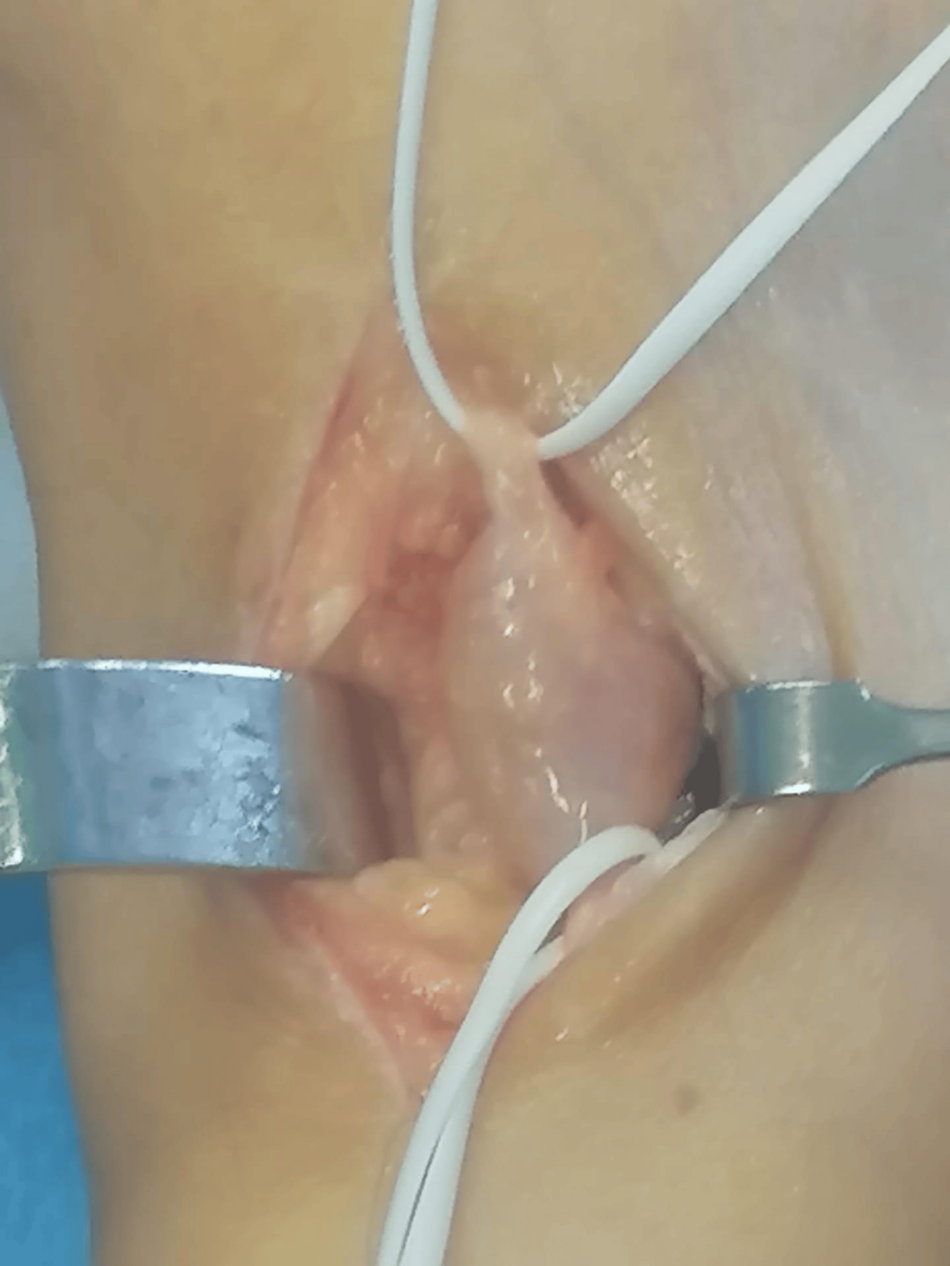
Image showing dorsal approach with isolating of the nerve and the tumor. White vessel loops refer to the proximal and distal aspects of the nerve

After the diagnosis of schwannoma was confirmed, the patient was advised to undergo surgical removal of the tumor. An anterior approach of the ankle was used, centered on the palpable mass. The tumor was isolated, and the proximal and distal ends of the lateral cutaneous branch of the superficial nerve were identified (Figure 2).

**Figure 3:**
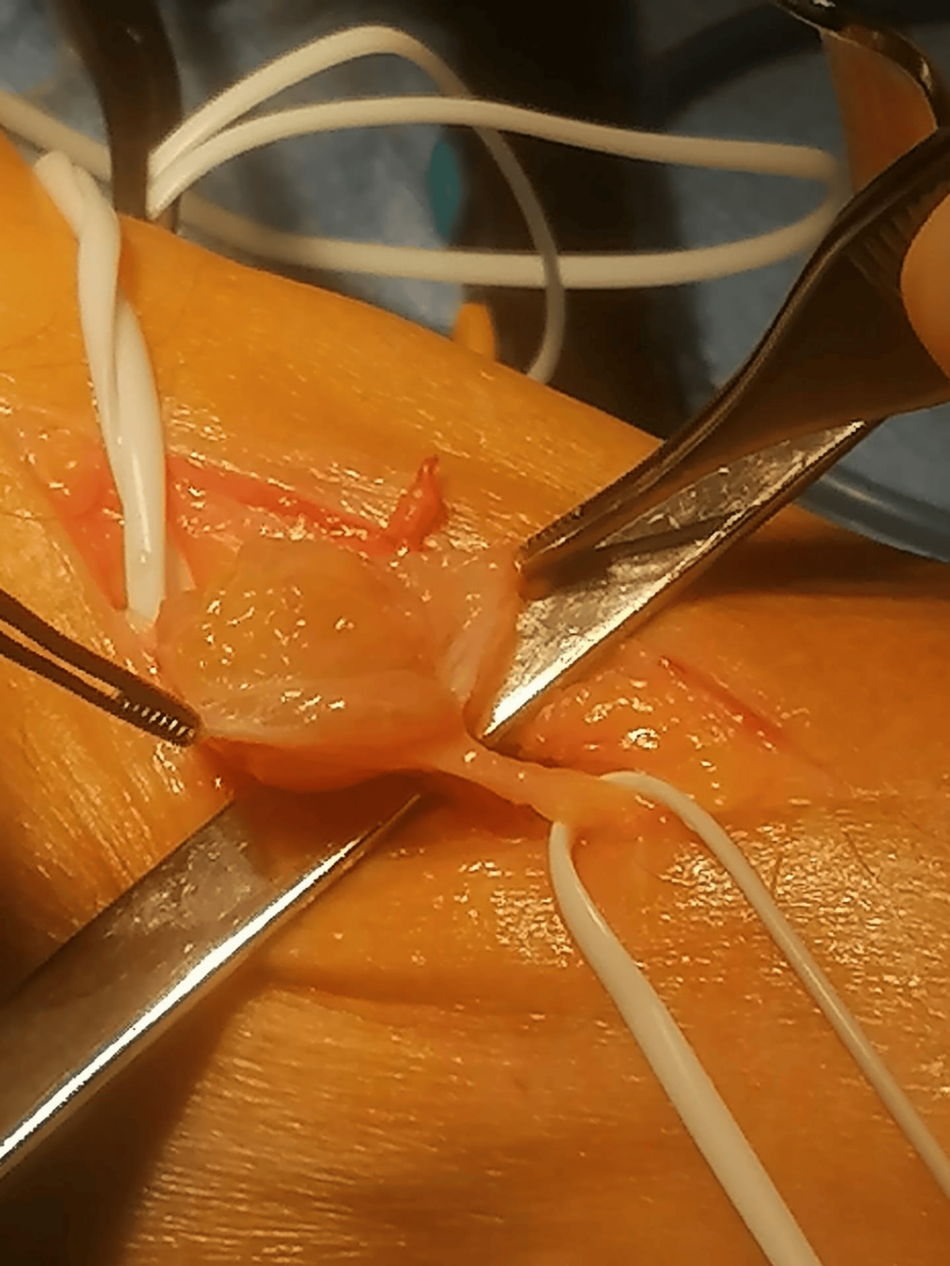
Image showing the opening of the nerve sheath referenced by the forceps

**Figure 4:**
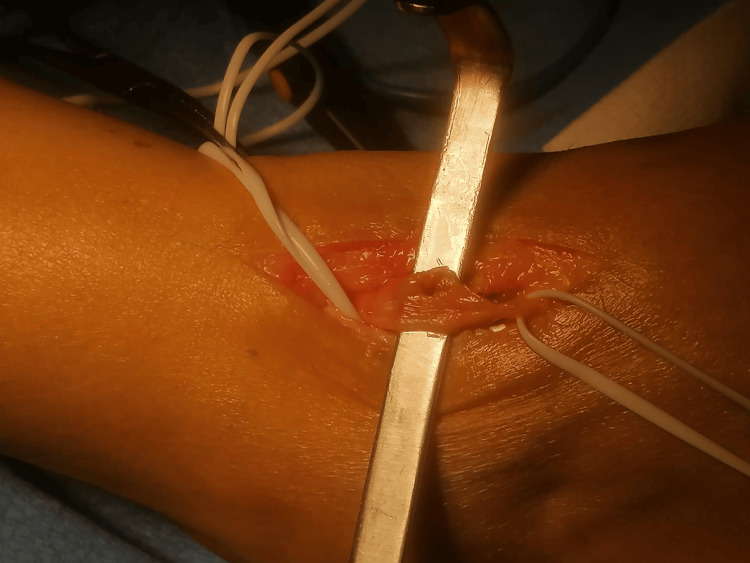
Image showing the intact nerve after the removal of the schwannoma

**Figure 5:**
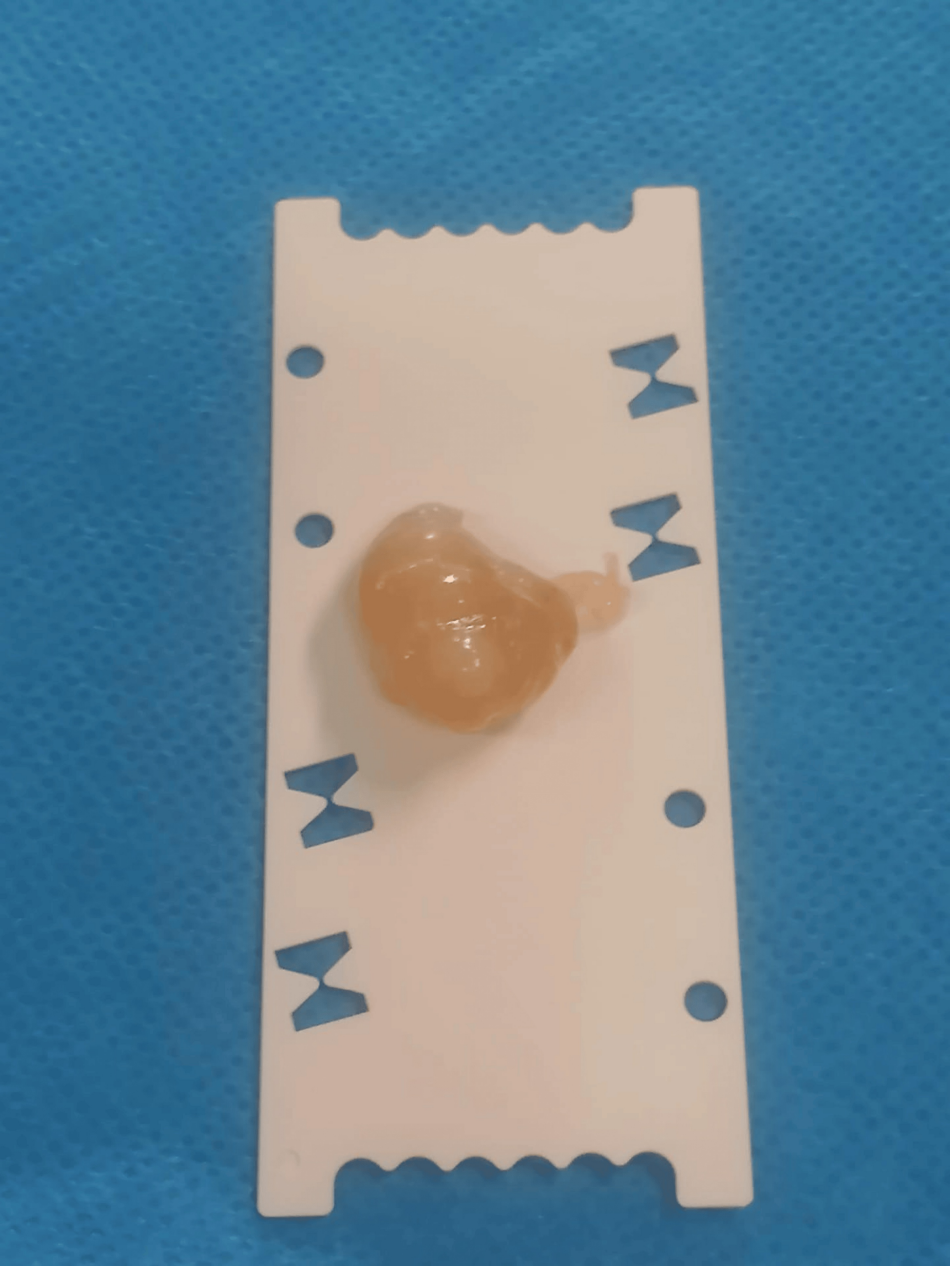
Tumor after excision

The nerve sheath was sharply opened (Figure 3), allowing for the careful isolation and removal of the tumor without causing additional nerve damage (Figures 4-5).

**Figure 6:**
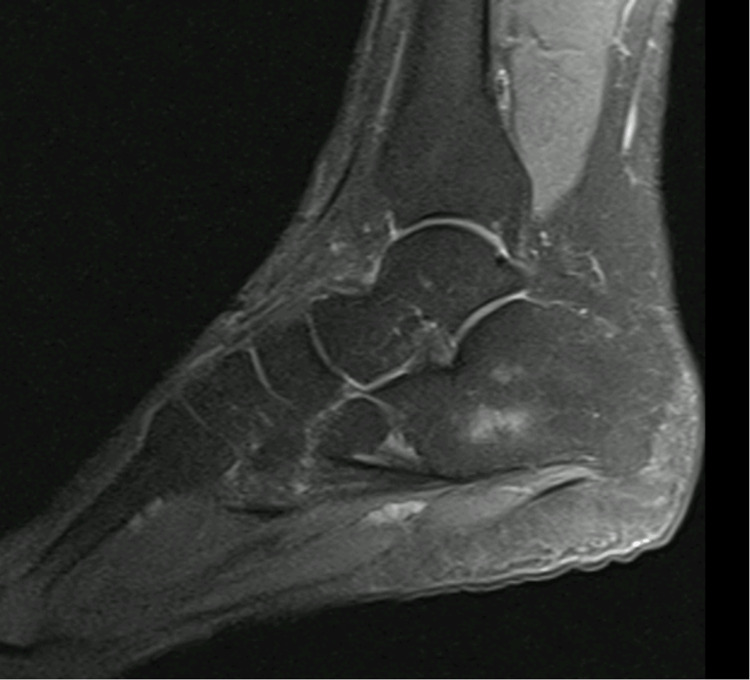
MRI image showing complete removal of the schwannoma

The histopathological examination of the specimen confirmed a schwannoma with no component of malignancy. At the four-week follow-up, the patient was asymptomatic without the neurologic deficit, and the two-month control MRI confirmed the complete removal of the schwannoma (Figure 6).

## Discussion

The schwannoma, or neurilemmoma, is the most common nerve sheath tumor, with 80-90% of cases occurring in the cerebellopontine angle [9]. The occurrence in peripheral nerves of the foot is rare, and when it occurs in the peripheral nerves, it tends to affect large nerves [6,11]. There is no difference in prevalence between men and women, with a peak age of diagnosis in the fourth decade of life [12].

Schwannomas are benign tumors, but malignant transformation can occur, with less than 1% of cases reported in the foot and ankle [13]. Normally, it is a single lesion but can also be associated with NF2 and, in this case, present with multiple lesions, a phenomenon called schwannomatosis [12,14].

Clinically, the schwannoma will present as a slow-growth mass without involving the nerve, and the symptoms will arise from the nerve compression with pain followed by paresthesia, hypoesthesia, and, in more severe compressions, motor deficit, and a Tinel sign might be present [11,12,15]. In the case presented, the patient complained mainly of paresthesia and pain associated with a palpable mass.

Concerning the diagnosis, both ultrasound and MRI are good options. An X-ray may be executed first to rule out any bony lesion or involvement that otherwise will be normal in the case of a schwannoma. Next, the MRI with gadolinium contrast will show an oval mass, encapsulated with the nerve entering one side and exiting the other side. The schwannoma will appear isointense in T1-weighted images and hyperintense in T2-weighted images [5,16]. In the case presented here, the authors opted to realize an MRI at the beginning without the need for an ultrasound afterward.

Once the diagnosis is final, the treatment consists of surgical excision of the tumor. The particularity about these tumors is that no fibers from the nerve are involved by the tumor. This fact is of utter importance for the surgeon because it will allow for the complete excision with no damage to the nerve. The steps of the procedure are as follows: first, the surgeon approaches the mass and isolates the tumor, identifying the proximal and distal ends of the nerve affected. Then, the nerve sheath is sharply opened, and the capsulated tumor is carefully dissected from the nerve without damaging the latter [3,17].

## Conclusions

This paper presents a rare case of schwannoma involving the superficial peroneal nerve, successfully treated through surgical excision. It is crucial for orthopedic surgeons to be well-versed in this pathology, as its diagnosis can easily be missed. In cases where a neurogenic tumor is clinically suspected, an MRI should be requested for confirmation. Once the diagnosis is established, surgical excision of the tumor is recommended. Meticulous dissection is essential to avoid nerve injury and to ensure complete removal of the mass.

Proper knowledge and surgical technique are vital for achieving the excellent prognosis typically associated with schwannomas when appropriately managed.
